# Supplementary material for: Lipidomic Profiling Reveals Biological Differences between Tumors of Self-Identified African Americans and Non-Hispanic Whites with Cancer
Source: Cancers (Basel). 2023 Apr 11;15(8):2238. doi: 10.3390/cancers15082238 (PMC10136787; doi:10.3390/cancers15082238)
Supplement: Supplementary file 1 [file cancers-15-02238-s001.zip › cancers-2235421-supplementary.pdf]

	LUAD	ceramide			monohexosylceramide			sphingomyelin			lactosylceramide		
acyl chain-length	***comparison***	***means diff.***	***p***	***p-adj. (q)***	***means diff.***	***p***	***p-adj. (q)***	***means diff.***	***p***	***p-adj. (q)***	***means diff.***	***p***	***p-adj. (q)***
14:0	*unin. vs tumor*	0.1	8.9E-01	7.2E-01	0.1	9.3E-01	7.2E-01	0.0	9.9E-01	1.0E+00	0.1	9.5E-01	9.3E-01
16:0	*unin. vs tumor*	2.1	**9.8E-03**	**2.4E-02**	2.6	**1.5E-05**	**3.6E-05**	2.9	3.6E-01	1.0E+00	2.4	**3.1E-03**	**3.0E-02**
18:1	*unin. vs tumor*	0.0	9.8E-01	7.2E-01	0.1	9.2E-01	7.2E-01	0.2	9.6E-01	1.0E+00	0.1	9.4E-01	9.3E-01
18:0	*unin. vs tumor*	0.1	8.7E-01	7.2E-01	0.0	9.6E-01	7.2E-01	0.8	8.1E-01	1.0E+00	0.1	8.6E-01	9.3E-01
20:0	*unin. vs tumor*	0.3	7.2E-01	7.2E-01	0.0	9.8E-01	7.2E-01	2.1	5.2E-01	1.0E+00	0.0	9.6E-01	9.3E-01
22:0	*unin. vs tumor*	1.4	8.5E-02	1.6E-01	0.8	2.0E-01	3.6E-01	0.4	8.9E-01	1.0E+00	0.4	5.9E-01	9.3E-01
24:1	*unin. vs tumor*	6.9	**1.0E-15**	**1.0E-15**	6.2	**1.0E-15**	**1.0E-15**	2.5	4.3E-01	1.0E+00	1.3	9.5E-02	4.1E-01
24:0	*unin. vs tumor*	3.6	**1.1E-05**	**4.1E-05**	3.3	**9.2E-08**	**3.4E-07**	0.6	8.6E-01	1.0E+00	1.2	1.3E-01	4.1E-01
26:1	*unin. vs tumor*	0.3	7.3E-01	7.2E-01	0.2	6.8E-01	7.2E-01	-0.5	8.8E-01	1.0E+00	0.0	9.5E-01	9.3E-01
26:0	*unin. vs tumor*	0.1	8.5E-01	7.2E-01	0.1	9.2E-01	7.2E-01	-0.1	9.8E-01	1.0E+00	0.0	9.9E-01	9.3E-01

	LUAD	LCBs		
species	***comparison***	***means diff.***	***p***	***p-adj. (q)***
d18:1 So	*unin. vs tumor*	-0.03	9.7E-01	1.0E+00
d18:0 Sa	*unin. vs tumor*	0.07	7.6E-01	1.0E+00
d18:1 So1P	*unin. vs tumor*	0.03	9.0E-01	1.0E+00
d18:0 Sa1P	*unin. vs tumor*	0.00	9.9E-01	1.0E+00

Supplemental Table S1. Uncorrected (p) and FDR/BKY-adjusted (Q=0.05%) p-values (q) for unin. versus tumor comparisons with LUAD tissues. For this analysis, data from AA and NHW were combined. Statistically significant values are in bold text. Positive means diff. values indicate corresponding lipid species is higher in the tumor than in the unin. tissue. The lower limit of p- and q-value calculations were set to 1.0E-15 (Prism), so no values are reported lower than this limit. Alterations considered significant (α ≤ 0.05, q ≤ 0.05) are in bold text. Means differences (means diff.) were calculated following ANOVA analysis (Prism).

	EEC	ceramide			monohexosylceramide			sphingomyelin			lactosylceramide		
acyl chain-length	***comparison***	***means diff.***	***p***	***p-adj. (q)***	***means diff.***	***p***	***p-adj. (q)***	***means diff.***	***p***	***p-adj. (q)***	***means diff.***	***p***	***p-adj. (q)***
14:0	*unin. vs tumor*	0.2	7.3E-01	5.1E-01	0.4	6.1E-01	5.2E-01	4.4	**1.6E-04**	**2.8E-04**	0.2	8.4E-01	7.1E-01
16:0	*unin. vs tumor*	1.7	**1.1E-02**	**1.7E-02**	7.4	**1.0E-15**	**1.0E-15**	3.5	**2.8E-03**	**2.9E-03**	11.7	**1.0E-15**	**1.0E-15**
18:1	*unin. vs tumor*	0.0	9.8E-01	6.2E-01	0.2	8.2E-01	5.2E-01	-0.2	8.7E-01	5.0E-01	0.3	7.2E-01	7.1E-01
18:0	*unin. vs tumor*	0.3	6.2E-01	4.9E-01	0.2	8.0E-01	5.2E-01	-1.6	1.5E-01	1.3E-01	0.1	9.1E-01	7.1E-01
20:0	*unin. vs tumor*	0.7	2.7E-01	3.4E-01	0.3	6.9E-01	5.2E-01	0.1	9.5E-01	5.0E-01	0.1	8.8E-01	7.1E-01
22:0	*unin. vs tumor*	3.7	**2.2E-08**	**4.6E-08**	3.5	**4.7E-05**	**7.5E-05**	3.9	**7.2E-04**	**9.5E-04**	0.9	3.2E-01	5.8E-01
24:1	*unin. vs tumor*	8.4	**1.0E-15**	**1.0E-15**	4.6	**1.7E-07**	**3.5E-07**	7.4	**2.7E-10**	**7.2E-10**	2.5	**3.6E-03**	**8.9E-03**
24:0	*unin. vs tumor*	11.0	**1.0E-15**	**1.0E-15**	12.7	**1.0E-15**	**1.0E-15**	9.6	**1.0E-15**	**5.0E-15**	3.6	**3.3E-05**	**1.2E-04**
26:1	*unin. vs tumor*	0.4	5.1E-01	4.9E-01	0.3	7.4E-01	5.2E-01	1.1	3.4E-01	2.2E-01	0.1	9.4E-01	7.1E-01
26:0	*unin. vs tumor*	0.4	5.6E-01	4.9E-01	0.5	5.2E-01	5.2E-01	1.4	2.2E-01	1.7E-01	0.0	9.7E-01	7.1E-01

	EEC	LCBs		
species	***comparison***	***means diff.***	***p***	***p-adj. (q)***
d18:1 So	*unin. vs tumor*	174.69	**2.2E-07**	**6.9E-07**
d18:0 Sa	*unin. vs tumor*	14.30	**9.6E-09**	**2.0E-08**
d18:1 So1P	*unin. vs tumor*	1.38	7.5E-02	1.6E-01
d18:0 Sa1P	*unin. vs tumor*	0.85	2.4E-01	2.5E-01

Supplemental Table S2. Uncorrected (p) and FDR/BKY-adjusted (Q=0.05%) p-values (q) for unin. versus tumor comparisons with EEC tissues. For this analysis, data from AA and NHW were combined. Statistically significant values are in bold text. Positive means diff. values indicate corresponding lipid species is higher in the tumor than in the unin. tissue. The lower limit of p- and q-value calculations were set to 1.0E-15 (Prism), so no values are reported lower than this limit. Alterations considered significant (α ≤ 0.05, q ≤ 0.05) are in bold text. Means differences (means diff.) were calculated following ANOVA analysis (Prism).

acyl chain-length	COAD comparison	ceramide means diff.	ceramide p	ceramide p-adj. (q)	monohexosylceramide means diff.	monohexosylceramide p	monohexosylceramide p-adj. (q)	sphingomyelin means diff.	sphingomyelin p	sphingomyelin p-adj. (q)	lactosylceramide means diff.	lactosylceramide p	lactosylceramide p-adj. (q)
14:0	*unin. vs tumor*	0.2	7.8E-01	7.3E-01	0.2	7.2E-01	7.3E-01	2.8	**9.3E-03**	**1.5E-02**	0.1	9.3E-01	8.4E-01
16:0	*unin. vs tumor*	2.3	**2.1E-03**	5.2E-03	4.9	**1.0E-15**	**1.0E-15**	3.7	**6.7E-04**	**2.1E-03**	15.9	**1.0E-15**	**1.0E-15**
18:1	*unin. vs tumor*	0.0	9.8E-01	7.3E-01	0.1	8.7E-01	7.3E-01	-0.4	7.3E-01	5.7E-01	0.0	9.9E-01	8.4E-01
18:0	*unin. vs tumor*	0.0	9.9E-01	7.3E-01	0.0	9.9E-01	7.3E-01	-3.9	**2.8E-04**	**1.8E-03**	0.1	9.6E-01	8.4E-01
20:0	*unin. vs tumor*	0.0	9.9E-01	7.3E-01	0.0	1.0E+00	7.3E-01	-3.4	**1.6E-03**	**3.3E-03**	0.0	9.9E-01	8.4E-01
22:0	*unin. vs tumor*	1.2	1.1E-01	2.0E-01	1.2	**3.6E-02**	6.6E-02	-2.0	6.9E-02	8.6E-02	0.8	4.6E-01	8.4E-01
24:1	*unin. vs tumor*	7.2	**1.0E-15**	**1.0E-15**	2.3	**5.4E-05**	**1.3E-04**	0.8	4.4E-01	3.9E-01	3.3	**2.4E-03**	**1.0E-02**
24:0	*unin. vs tumor*	4.5	**8.7E-10**	**3.2E-09**	6.1	**1.0E-15**	**1.0E-15**	0.9	4.2E-01	3.9E-01	2.3	**2.9E-02**	**8.0E-02**
26:1	*unin. vs tumor*	0.2	7.4E-01	7.3E-01	0.1	8.1E-01	7.3E-01	-0.1	9.1E-01	6.1E-01	0.1	9.4E-01	8.4E-01
26:0	*unin. vs tumor*	0.2	8.0E-01	7.3E-01	0.2	6.9E-01	7.3E-01	0.0	9.7E-01	6.1E-01	0.0	9.9E-01	8.4E-01

species	COAD comparison	LCBs means diff.	LCBs p	LCBs p-adj. (q)
d18:1 So	*unin. vs tumor*	4.75	**5.7E-04**	**1.7E-03**
d18:0 Sa	*unin. vs tumor*	6.95	**6.5E-06**	**2.0E-05**
d18:1 So1P	*unin. vs tumor*	0.03	6.3E-01	6.3E-01
d18:0 Sa1P	*unin. vs tumor*	0.08	1.9E-01	3.4E-01

Supplemental Table S3. Uncorrected (p) and FDR/BKY-adjusted (Q=0.05%) p-values (q) for unin. versus tumor comparisons with COAD tissues. For this analysis, data from AA and NHW were combined. Statistically significant values are in bold text. Positive means diff. values indicate corresponding lipid species is higher in the tumor than in the unin. tissue. The lower limit of p- and q-value calculations were set to 1.0E-15 (Prism), so no values are reported lower than this limit. Alterations considered significant ($\alpha \leq 0.05$, $q \leq 0.05$) are in bold text. Means differences (means diff.) were calculated following ANOVA analysis (Prism).

acyl chain-length	HCC *comparison*	ceramide *means diff.*	*p*	*p-adj. (q)*	monohexosylceramide *means diff.*	*p*	*p-adj. (q)*	sphingomyelin *means diff.*	*p*	*p-adj. (q)*	lactosylceramide *means diff.*	*p*	*p-adj. (q)*
14:0	*unin. vs tumor*	0.1	9.6E-01	1.0E+00	0.0	9.9E-01	8.4E-01	-1.1	6.7E-01	1.0E+00	-0.1	9.4E-01	1.0E+00
16:0	*unin. vs tumor*	1.3	6.1E-01	1.0E+00	0.1	9.2E-01	8.4E-01	-0.4	8.6E-01	1.0E+00	-2.6	**1.9E-02**	9.7E-02
18:1	*unin. vs tumor*	0.5	8.5E-01	1.0E+00	0.0	1.0E+00	8.4E-01	0.1	9.6E-01	1.0E+00	0.1	9.5E-01	1.0E+00
18:0	*unin. vs tumor*	0.0	9.9E-01	1.0E+00	0.0	9.9E-01	8.4E-01	-0.5	8.4E-01	1.0E+00	0.1	9.6E-01	1.0E+00
20:0	*unin. vs tumor*	-0.5	8.5E-01	1.0E+00	0.0	9.6E-01	8.4E-01	-3.0	2.3E-01	7.0E-01	-0.1	9.2E-01	1.0E+00
22:0	*unin. vs tumor*	-0.3	9.1E-01	1.0E+00	-2.4	**3.7E-03**	**1.6E-02**	-4.5	6.7E-02	7.0E-01	-2.6	**1.7E-02**	9.7E-02
24:1	*unin. vs tumor*	4.4	8.7E-02	9.2E-01	0.0	9.9E-01	8.4E-01	-2.8	2.7E-01	7.0E-01	-1.0	3.6E-01	9.4E-01
24:0	*unin. vs tumor*	0.6	8.1E-01	1.0E+00	-3.8	**4.2E-06**	**3.6E-05**	-3.0	2.2E-01	7.0E-01	-2.0	6.8E-02	2.4E-01
26:1	*unin. vs tumor*	0.4	8.8E-01	1.0E+00	0.1	9.4E-01	8.4E-01	0.1	9.6E-01	1.0E+00	0.0	9.9E-01	1.0E+00
26:0	*unin. vs tumor*	0.1	9.7E-01	1.0E+00	0.0	9.6E-01	8.4E-01	-0.4	8.6E-01	1.0E+00	0.0	9.9E-01	1.0E+00

species	HCC *comparison*	LCBs *means diff.*	*p*	*p-adj. (q)*
d18:1 So	*unin. vs tumor*	-8.70	**6.9E-03**	**2.7E-02**
d18:0 Sa	*unin. vs tumor*	-5.49	**1.6E-03**	**4.9E-03**
d18:1 So1P	*unin. vs tumor*	-0.10	**2.6E-02**	5.2E-02
d18:0 Sa1P	*unin. vs tumor*	0.00	1.0E+00	1.0E+00

Supplemental Table S4. Uncorrected (p) and FDR/BKY-adjusted (Q=0.05%) p-values (q) for unin. versus tumor comparisons with HCC tissues. For this analysis, data from AA and NHW were combined. Statistically significant values are in bold text. Positive means diff. values indicate corresponding lipid species is higher in the tumor than in the unin. tissue. The lower limit of p- and q-value calculations were set to 1.0E-15 (Prism), so no values are reported lower than this limit. Alterations considered significant (α ≤ 0.05, q ≤ 0.05) are in bold text. Means differences (means diff.) were calculated following ANOVA analysis (Prism).

	HNSCC	ceramide			monohexosylceramide			sphingomyelin			lactosylceramide		
acyl chain-length	***comparison***	***means diff.***	***p***	***p-adj. (q)***	***means diff.***	***p***	***p-adj. (q)***	***means diff.***	***p***	***p-adj. (q)***	***means diff.***	***p***	***p-adj. (q)***
14:0	*unin. vs tumor*	0.8	5.1E-01	2.9E-01	0.1	9.3E-01	8.3E-01	5.3	9.3E-02	3.2E-01	0.3	9.2E-01	7.0E-01
16:0	*unin. vs tumor*	7.6	**7.0E-11**	**1.8E-10**	5.6	**1.4E-03**	**6.1E-03**	-3.4	2.8E-01	5.9E-01	38.1	**1.0E-15**	**1.0E-15**
18:1	*unin. vs tumor*	0.1	9.5E-01	5.0E-01	0.0	9.9E-01	8.3E-01	-1.8	5.8E-01	8.8E-01	0.2	9.5E-01	7.0E-01
18:0	*unin. vs tumor*	0.9	4.4E-01	2.9E-01	0.5	7.8E-01	8.2E-01	-6.1	5.4E-02	2.8E-01	0.5	8.7E-01	7.0E-01
20:0	*unin. vs tumor*	2.0	8.0E-02	7.0E-02	0.8	6.5E-01	8.2E-01	-1.4	6.7E-01	8.8E-01	0.2	9.5E-01	7.0E-01
22:0	*unin. vs tumor*	3.5	**2.4E-03**	**2.5E-03**	-0.9	6.0E-01	8.2E-01	0.5	8.7E-01	9.1E-01	4.7	1.3E-01	2.5E-01
24:1	*unin. vs tumor*	5.0	**1.4E-05**	**1.8E-05**	3.2	6.6E-02	1.9E-01	0.9	7.9E-01	9.1E-01	23.0	**6.7E-13**	**2.5E-12**
24:0	*unin. vs tumor*	11.2	**1.0E-15**	**1.0E-15**	6.1	**4.7E-04**	**3.9E-03**	4.9	1.2E-01	3.2E-01	13.5	**1.7E-05**	**4.1E-05**
26:1	*unin. vs tumor*	1.0	3.8E-01	2.9E-01	3.0	8.9E-02	1.9E-01	6.3	**4.9E-02**	2.8E-01	0.8	7.9E-01	7.0E-01
26:0	*unin. vs tumor*	6.7	**7.1E-09**	**1.2E-08**	0.7	6.9E-01	8.2E-01	1.4	6.7E-01	8.8E-01	0.2	9.5E-01	7.0E-01

	HNSCC	LCBs		
species	***comparison***	***means diff.***	***p***	***p-adj. (q)***
d18:1 So	*unin. vs tumor*	-10.66	5.5E-02	2.3E-01
d18:0 Sa	*unin. vs tumor*	-1.17	6.4E-01	1.0E+00
d18:1 So1P	*unin. vs tumor*	-0.49	1.5E-01	3.1E-01
d18:0 Sa1P	*unin. vs tumor*	0.01	9.9E-01	1.0E+00

Supplemental Table S5. Uncorrected (p) and FDR/BKY-adjusted (Q=0.05) p-values (q) for unin. versus tumor comparisons with HNSCC tissues. For this analysis, data from AA and NHW were combined. Statistically significant values are in bold text. Means diff. values that are positive indicate that the corresponding lipid species is higher in the tumor than in the unin. tissue. The lower limit of p- and q-value calculations were set to 1.0E-15 (Prism), so no values are reported lower than this limit. Alterations considered significant (α ≤ 0.05, q ≤ 0.05) are in bold text. Means differences (means diff.) were calculated following ANOVA analysis (Prism).

	LUAD AA	ceramide			monohexosylceramide			sphingomyelin			lactosylceramide		
acyl chain-length	***comparison***	***means diff.***	***p***	***p-adj. (q)***	***means diff.***	***p***	***p-adj. (q)***	***means diff.***	***p***	***p-adj. (q)***	***means diff.***	***p***	***p-adj. (q)***
14:0	AA unin. vs AA tumor	0.06	9.6E-01	9.4E-01	0.05	9.6E-01	8.4E-01	-0.31	9.7E-01	1.0E+00	-0.01	1.0E+00	1.0E+00
16:0	AA unin. vs AA tumor	0.99	3.9E-01	9.1E-01	2.30	**3.2E-02**	8.9E-02	-1.47	8.4E-01	1.0E+00	2.72	**1.0E-02**	1.1E-01
18:1	AA unin. vs AA tumor	-0.04	9.7E-01	9.4E-01	0.03	9.8E-01	8.4E-01	0.17	9.8E-01	1.0E+00	0.03	9.8E-01	1.0E+00
18:0	AA unin. vs AA tumor	0.00	1.0E+00	9.4E-01	-0.02	9.8E-01	8.4E-01	-1.00	8.9E-01	1.0E+00	0.07	9.5E-01	1.0E+00
20:0	AA unin. vs AA tumor	0.13	9.1E-01	9.4E-01	0.00	1.0E+00	8.4E-01	3.72	6.2E-01	1.0E+00	0.00	1.0E+00	1.0E+00
22:0	AA unin. vs AA tumor	1.13	3.3E-01	9.1E-01	1.03	3.4E-01	7.0E-01	-4.28	5.6E-01	1.0E+00	0.23	8.2E-01	1.0E+00
24:1	AA unin. vs AA tumor	4.99	**1.9E-05**	**1.8E-04**	6.42	**6.4E-09**	**5.3E-08**	0.91	9.0E-01	1.0E+00	0.86	4.1E-01	1.0E+00
24:0	AA unin. vs AA tumor	2.09	6.8E-02	3.2E-01	3.62	**8.0E-04**	**3.4E-03**	0.28	9.7E-01	1.0E+00	0.58	5.8E-01	1.0E+00
26:1	AA unin. vs AA tumor	0.11	9.2E-01	9.4E-01	0.29	7.9E-01	8.4E-01	-0.84	9.1E-01	1.0E+00	0.02	9.9E-01	1.0E+00
26:0	AA unin. vs AA tumor	0.08	9.5E-01	9.4E-01	0.06	9.6E-01	8.4E-01	0.21	9.8E-01	1.0E+00	0.00	1.0E+00	1.0E+00

	LUAD AA	sphingoid bases		
species	***comparison***	***means diff.***	***p***	***p-adj. (q)***
d18:1 So	AA unin. vs AA tumor	-1.57	**4.8E-02**	2.0E-01
d18:0 Sa	AA unin. vs AA tumor	-0.12	6.1E-01	1.0E+00
d18:1 So1P	AA unin. vs AA tumor	0.04	8.7E-01	1.0E+00
d18:0 Sa1P	AA unin. vs AA tumor	0.00	9.8E-01	1.0E+00

Supplemental Table S6. Uncorrected (p) and FDR/BKY-adjusted (Q=0.05) p-values (q) for unin. versus tumor comparisons with LUAD tissues from AA males. Means diff. values that are positive indicate that the corresponding lipid species is higher in the tumor than in the unin. tissue. The lower limit of p- and q-value calculations were set to 1.0E-15 (Prism), so no values are reported lower than this limit. Alterations considered significant (α ≤ 0.05, q ≤ 0.05) are in bold text. Means differences (means diff.) were calculated following ANOVA analysis (Prism).

	EEC AA	ceramide			monohexosylceramide			sphingomyelin			lactosylceramide		
acyl chain-length	***comparison***	***means diff.***	***p***	***p-adj. (q)***	***means diff.***	***p***	***p-adj. (q)***	***means diff.***	***p***	***p-adj. (q)***	***means diff.***	***p***	***p-adj. (q)***
14:0	AA unin. vs AA tumor	0.19	8.4E-01	7.0E-01	0.42	6.3E-01	5.4E-01	5.80	**5.6E-05**	**9.8E-05**	0.25	8.5E-01	8.3E-01
16:0	AA unin. vs AA tumor	1.24	1.9E-01	3.5E-01	5.36	**4.1E-09**	**1.3E-08**	4.28	**2.6E-03**	**3.5E-03**	10.73	**2.5E-14**	**2.1E-13**
18:1	AA unin. vs AA tumor	0.01	9.9E-01	7.3E-01	0.16	8.5E-01	5.4E-01	-0.12	9.3E-01	4.9E-01	0.33	8.0E-01	8.3E-01
18:0	AA unin. vs AA tumor	0.26	7.8E-01	7.0E-01	0.22	8.0E-01	5.4E-01	-1.52	2.8E-01	2.5E-01	0.20	8.8E-01	8.3E-01
20:0	AA unin. vs AA tumor	0.68	4.7E-01	6.9E-01	0.45	6.0E-01	5.4E-01	0.80	5.7E-01	3.3E-01	0.17	9.0E-01	8.3E-01
22:0	AA unin. vs AA tumor	3.56	**2.0E-04**	**4.9E-04**	3.39	**1.3E-04**	**2.1E-04**	4.03	**4.6E-03**	**4.8E-03**	1.04	4.2E-01	8.3E-01
24:1	AA unin. vs AA tumor	6.96	**4.4E-12**	**1.6E-11**	4.68	**2.1E-07**	**4.3E-07**	6.17	**1.9E-05**	**5.0E-05**	2.32	7.4E-02	2.1E-01
24:0	AA unin. vs AA tumor	11.41	**1.0E-15**	**1.0E-15**	12.55	**1.0E-15**	**1.0E-15**	8.26	**2.0E-08**	**1.0E-07**	4.45	**7.2E-04**	**3.0E-03**
26:1	AA unin. vs AA tumor	0.30	7.5E-01	7.0E-01	0.22	8.0E-01	5.4E-01	0.89	5.3E-01	3.3E-01	0.06	9.6E-01	8.3E-01
26:0	AA unin. vs AA tumor	0.17	8.6E-01	7.0E-01	0.29	7.4E-01	5.4E-01	0.92	5.1E-01	3.3E-01	0.02	9.8E-01	8.3E-01

	EEC AA	sphingoid bases		
species	***comparison***	***means diff.***	***p***	***p-adj. (q)***
d18:1 So	AA unin. vs AA tumor	245.29	**3.6E-04**	**1.4E-03**
d18:0 Sa	AA unin. vs AA tumor	13.42	**2.3E-05**	**6.8E-05**
d18:1 So1P	AA unin. vs AA tumor	0.83	7.9E-01	9.5E-01
d18:0 Sa1P	AA unin. vs AA tumor	0.18	9.5E-01	9.5E-01

Supplemental Table S7. Uncorrected (p) and FDR/BKY-adjusted (Q=0.05) p-values (q) for unin. versus tumor comparisons with EEC tissues from AA females. Means diff. values that are positive indicate that the corresponding lipid species is higher in the tumor than in the unin. tissue. The lower limit of p- and q-value calculations were set to 1.0E-15 (Prism), so no values are reported lower than this limit. Alterations considered significant (α ≤ 0.05, q ≤ 0.05) are in bold text. Means differences (means diff.) were calculated following ANOVA analysis (Prism).

	COAD AA	ceramide			monohexosylceramide			sphingomyelin			lactosylceramide		
acyl chain-length	***comparison***	***means diff.***	***p***	***p-adj. (q)***	***means diff.***	***p***	***p-adj. (q)***	***means diff.***	***p***	***p-adj. (q)***	***means diff.***	***p***	***p-adj. (q)***
14:0	AA unin. vs AA tumor	0.23	8.4E-01	8.4E-01	0.27	7.3E-01	7.3E-01	3.32	**3.5E-02**	8.2E-02	0.08	9.6E-01	9.4E-01
16:0	AA unin. vs AA tumor	2.39	**3.3E-02**	9.2E-02	5.17	**1.1E-10**	**3.9E-10**	5.57	**4.1E-04**	**3.9E-03**	18.08	**1.0E-15**	**1.0E-15**
18:1	AA unin. vs AA tumor	0.03	9.8E-01	8.4E-01	0.13	8.7E-01	7.3E-01	-0.31	8.4E-01	9.2E-01	0.06	9.7E-01	9.4E-01
18:0	AA unin. vs AA tumor	0.03	9.8E-01	8.4E-01	0.00	1.0E+00	7.3E-01	-3.93	**1.3E-02**	5.9E-02	0.07	9.7E-01	9.4E-01
20:0	AA unin. vs AA tumor	0.00	1.0E+00	8.4E-01	-0.01	9.9E-01	7.3E-01	-3.40	**3.1E-02**	**8.2E-02**	0.02	9.9E-01	9.4E-01
22:0	AA unin. vs AA tumor	1.20	2.8E-01	6.0E-01	0.90	2.5E-01	4.7E-01	-2.54	1.1E-01	2.0E-01	0.84	6.1E-01	9.4E-01
24:1	AA unin. vs AA tumor	9.06	**3.0E-15**	**2.8E-14**	2.21	**5.1E-03**	**1.3E-02**	0.58	7.1E-01	9.2E-01	3.68	**2.5E-02**	1.2E-01
24:0	AA unin. vs AA tumor	5.25	**3.4E-06**	**1.4E-05**	5.83	**4.1E-13**	**3.0E-12**	1.08	4.9E-01	7.7E-01	2.52	1.3E-01	4.0E-01
26:1	AA unin. vs AA tumor	0.35	7.5E-01	8.4E-01	0.14	8.5E-01	7.3E-01	-0.09	9.5E-01	9.2E-01	0.08	9.6E-01	9.4E-01
26:0	AA unin. vs AA tumor	0.18	8.7E-01	8.4E-01	0.15	8.5E-01	7.3E-01	-0.04	9.8E-01	9.2E-01	0.02	9.9E-01	9.4E-01

	COAD AA	sphingoid bases		
species	***comparison***	***means diff.***	***p***	***p-adj. (q)***
d18:1 So	AA unin. vs AA tumor	4.27	**1.5E-02**	**1.6E-02**
d18:0 Sa	AA unin. vs AA tumor	6.59	**8.8E-04**	**1.9E-03**
d18:1 So1P	AA unin. vs AA tumor	0.04	9.8E-01	6.9E-01
d18:0 Sa1P	AA unin. vs AA tumor	0.16	9.3E-01	6.9E-01

Supplemental Table S8. Uncorrected (p) and FDR/BKY-adjusted (Q=0.05) p-values (q) for unin. versus tumor comparisons with COAD tissues from AA males. Means diff. values that are positive indicate that the corresponding lipid species is higher in the tumor than in the unin. tissue. The lower limit of p- and q-value calculations were set to 1.0E-15 (Prism), so no values are reported lower than this limit. Alterations considered significant ($\alpha \leq 0.05$, $q \leq 0.05$) are in bold text. Means differences (means diff.) were calculated following ANOVA analysis (Prism).

	HCC AA	ceramide			monohexosylceramide			sphingomyelin			lactosylceramide		
acyl chain-length	***comparison***	***means diff.***	***p***	***p-adj. (q)***	***means diff.***	***p***	***p-adj. (q)***	***means diff.***	***p***	***p-adj. (q)***	***means diff.***	***p***	***p-adj. (q)***
14:0	AA unin. vs AA tumor	0.13	9.8E-01	1.0E+00	0.01	9.9E-01	9.4E-01	-4.23	3.9E-01	1.0E+00	-0.16	9.3E-01	9.4E-01
16:0	AA unin. vs AA tumor	0.73	8.8E-01	1.0E+00	-0.31	7.6E-01	9.4E-01	-1.49	7.6E-01	1.0E+00	-5.52	**1.8E-03**	**1.7E-02**
18:1	AA unin. vs AA tumor	0.08	9.9E-01	1.0E+00	-0.01	9.9E-01	9.4E-01	0.06	9.9E-01	1.0E+00	-0.27	8.8E-01	9.4E-01
18:0	AA unin. vs AA tumor	-0.23	9.6E-01	1.0E+00	-0.02	9.9E-01	9.4E-01	-1.13	8.2E-01	1.0E+00	0.07	9.7E-01	9.4E-01
20:0	AA unin. vs AA tumor	-0.53	9.1E-01	1.0E+00	-0.03	9.8E-01	9.4E-01	-3.51	4.8E-01	1.0E+00	-0.15	9.3E-01	9.4E-01
22:0	AA unin. vs AA tumor	1.66	7.2E-01	1.0E+00	-0.77	4.5E-01	9.4E-01	-8.35	9.2E-02	3.5E-01	-2.67	1.3E-01	4.0E-01
24:1	AA unin. vs AA tumor	2.01	6.7E-01	1.0E+00	-0.06	9.5E-01	9.4E-01	-8.15	9.9E-02	3.5E-01	-1.18	5.0E-01	9.4E-01
24:0	AA unin. vs AA tumor	7.59	1.1E-01	1.0E+00	-3.00	**3.6E-03**	**3.4E-02**	-8.34	9.2E-02	3.5E-01	-2.90	9.8E-02	4.0E-01
26:1	AA unin. vs AA tumor	0.48	9.2E-01	1.0E+00	0.05	9.6E-01	9.4E-01	-0.14	9.8E-01	1.0E+00	-0.02	9.9E-01	9.4E-01
26:0	AA unin. vs AA tumor	0.04	9.9E-01	1.0E+00	-0.02	9.9E-01	9.4E-01	-0.53	9.1E-01	1.0E+00	-0.02	9.9E-01	9.4E-01

	HCC AA	sphingoid bases		
species	***comparison***	***means diff.***	***p***	***p-adj. (q)***
d18:1 So	AA unin. vs AA tumor	-10.25	5.9E-02	2.5E-01
d18:0 Sa	AA unin. vs AA tumor	-8.12	**7.4E-03**	**1.5E-02**
d18:1 So1P	AA unin. vs AA tumor	-0.06	**2.4E-04**	**2.6E-04**
d18:0 Sa1P	AA unin. vs AA tumor	0.00	9.5E-01	1.0E+00

Supplemental Table S9. Uncorrected (p) and FDR/BKY-adjusted (Q=0.05) p-values (q) for unin. versus tumor comparisons with HCC tissues from AA males. Means diff. values that are positive indicate that the corresponding lipid species is higher in the tumor than in the unin. tissue. The lower limit of p- and q-value calculations were set to 1.0E-15 (Prism), so no values are reported lower than this limit. Alterations considered significant (α ≤ 0.05, q ≤ 0.05) are in bold text. Means differences (means diff.) were calculated following ANOVA analysis (Prism).

acyl chain-length	HNSCC AA comparison	ceramide means diff.	ceramide p	ceramide p-adj. (q)	monohexosylceramide means diff.	monohexosylceramide p	monohexosylceramide p-adj. (q)	sphingomyelin means diff.	sphingomyelin p	sphingomyelin p-adj. (q)	lactosylceramide means diff.	lactosylceramide p	lactosylceramide p-adj. (q)
14:0	AA unin. vs AA tumor	1.13	5.5E-01	3.2E-01	0.29	9.2E-01	8.3E-01	7.85	1.1E-01	3.5E-01	0.32	9.4E-01	7.0E-01
16:0	AA unin. vs AA tumor	9.31	**1.7E-06**	**4.4E-06**	11.38	**9.2E-05**	**3.9E-04**	-8.48	8.6E-02	3.5E-01	47.46	**1.0E-15**	**1.0E-15**
18:1	AA unin. vs AA tumor	0.02	9.9E-01	5.2E-01	0.03	9.9E-01	8.3E-01	-1.06	8.3E-01	9.9E-01	0.27	9.5E-01	7.0E-01
18:0	AA unin. vs AA tumor	1.28	5.0E-01	3.2E-01	0.87	7.6E-01	8.3E-01	-7.43	1.3E-01	3.5E-01	0.74	8.6E-01	7.0E-01
20:0	AA unin. vs AA tumor	3.02	1.1E-01	9.7E-02	1.54	5.9E-01	8.3E-01	0.46	9.3E-01	9.9E-01	0.37	9.3E-01	7.0E-01
22:0	AA unin. vs AA tumor	4.57	**1.6E-02**	**1.7E-02**	1.90	5.1E-01	8.3E-01	2.24	6.5E-01	9.9E-01	5.36	2.0E-01	3.6E-01
24:1	AA unin. vs AA tumor	7.16	**2.0E-04**	**2.6E-04**	5.99	**3.7E-02**	1.0E-01	0.34	9.4E-01	9.9E-01	23.03	**8.8E-08**	**3.2E-07**
24:0	AA unin. vs AA tumor	14.43	**7.6E-13**	**4.0E-12**	12.35	**2.3E-05**	**2.0E-04**	7.45	1.3E-01	3.5E-01	14.47	**6.0E-04**	**1.5E-03**
26:1	AA unin. vs AA tumor	1.36	4.7E-01	3.2E-01	5.19	7.0E-02	1.5E-01	6.20	2.1E-01	4.4E-01	1.11	7.9E-01	7.0E-01
26:0	AA unin. vs AA tumor	7.55	**8.9E-05**	**1.6E-04**	0.63	8.2E-01	8.3E-01	1.52	7.6E-01	9.9E-01	1.03	8.0E-01	7.0E-01

species	HNSCC AA comparison	sphingoid bases means diff.	sphingoid bases p	sphingoid bases p-adj. (q)
d18:1 So	AA unin. vs AA tumor	-5.75	1.9E-01	7.9E-01
d18:0 Sa	AA unin. vs AA tumor	-3.44	3.1E-01	9.7E-01
d18:1 So1P	AA unin. vs AA tumor	-0.13	2.1E-01	4.5E-01
d18:0 Sa1P	AA unin. vs AA tumor	-0.02	8.7E-01	9.1E-01

Supplemental Table S10. Uncorrected (p) and FDR/BKY-adjusted (Q=0.05) p-values (q) for unin. versus tumor comparisons with HNSCC tissues from AA males. Means diff. values that are positive indicate that the corresponding lipid species is higher in the tumor than in the unin. tissue. The lower limit of p- and q-value calculations were set to 1.0E-15 (Prism), so no values are reported lower than this limit. Alterations considered significant (α ≤ 0.05, q ≤ 0.05) are in bold text. Means differences (means diff.) were calculated following ANOVA analysis (Prism).

acyl chain-length	LUAD NHW comparison	ceramide means diff.	ceramide p	ceramide p-adj. (q)	monohexosylceramide means diff.	monohexosylceramide p	monohexosylceramide p-adj. (q)	sphingomyelin means diff.	sphingomyelin p	sphingomyelin p-adj. (q)	lactosylceramide means diff.	lactosylceramide p	lactosylceramide p-adj. (q)
14:0	NHW unin. vs NHW tumor	0.13	9.0E-01	7.1E-01	0.05	9.5E-01	7.2E-01	0.08	9.8E-01	1.0E+00	0.08	9.4E-01	1.0E+00
16:0	NHW unin. vs NHW tumor	2.72	**1.3E-02**	**3.3E-02**	2.83	**1.7E-04**	**4.1E-04**	5.32	6.6E-02	7.0E-01	2.20	**4.4E-02**	4.6E-01
18:1	NHW unin. vs NHW tumor	0.05	9.6E-01	7.1E-01	0.08	9.1E-01	7.2E-01	0.13	9.6E-01	1.0E+00	0.07	9.5E-01	1.0E+00
18:0	NHW unin. vs NHW tumor	0.21	8.5E-01	7.1E-01	0.06	9.3E-01	7.2E-01	1.69	5.6E-01	1.0E+00	0.18	8.7E-01	1.0E+00
20:0	NHW unin. vs NHW tumor	0.37	7.3E-01	7.1E-01	-0.02	9.8E-01	7.2E-01	1.09	7.1E-01	1.0E+00	0.06	9.6E-01	1.0E+00
22:0	NHW unin. vs NHW tumor	1.55	1.6E-01	2.9E-01	0.65	3.8E-01	7.1E-01	2.97	3.0E-01	1.0E+00	0.54	6.2E-01	1.0E+00
24:1	NHW unin. vs NHW tumor	8.04	**9.8E-13**	**7.2E-12**	6.04	**5.0E-15**	**3.9E-14**	3.39	2.4E-01	1.0E+00	1.61	1.4E-01	5.3E-01
24:0	NHW unin. vs NHW tumor	4.42	**6.3E-05**	**2.3E-04**	3.07	**4.6E-05**	**1.7E-04**	0.70	8.1E-01	1.0E+00	1.57	1.5E-01	5.3E-01
26:1	NHW unin. vs NHW tumor	0.38	7.3E-01	7.1E-01	0.22	7.7E-01	7.2E-01	-0.31	9.1E-01	1.0E+00	0.07	9.5E-01	1.0E+00
26:0	NHW unin. vs NHW tumor	0.19	8.6E-01	7.1E-01	0.07	9.3E-01	7.2E-01	-0.24	9.3E-01	1.0E+00	0.02	9.8E-01	1.0E+00

species	LUAD NHW comparison	sphingoid bases means diff.	p	p-adj. (q)
d18:1 So	NHW unin. vs NHW tumor	0.84	4.3E-01	1.0E+00
d18:0 Sa	NHW unin. vs NHW tumor	0.19	5.9E-01	1.0E+00
d18:1 So1P	NHW unin. vs NHW tumor	0.03	9.4E-01	1.0E+00
d18:0 Sa1P	NHW unin. vs NHW tumor	0.01	9.8E-01	1.0E+00

Supplemental Table S11. Uncorrected (p) and FDR/BKY-adjusted (Q=0.05) p-values (q) for comparisons of unin. vs tumor tissues from NHW males with LUAD. Means diff. values that are positive indicate that the corresponding lipid species is higher in the tumor than in the unin. tissue. The lower limit of p- and q-value calculations were set to 1.0E-15 (Prism), so no values are reported lower than this limit. Alterations considered significant ($\alpha \leq 0.05$, $q \leq 0.05$) are in bold text. Means differences (means diff.) were calculated following ANOVA analysis (Prism).

	EEC NHW	ceramide			monohexosylceramide			sphingomyelin			lactosylceramide		
acyl chain-length	***comparison***	***means diff.***	***p***	***p-adj. (q)***	***means diff.***	***p***	***p-adj. (q)***	***means diff.***	***p***	***p-adj. (q)***	***means diff.***	***p***	***p-adj. (q)***
14:0	NHW unin. vs NHW tumor	0.25	7.8E-01	6.1E-01	0.47	7.4E-01	5.5E-01	3.10	8.1E-02	4.5E-01	0.10	9.4E-01	1.0E+00
16:0	NHW unin. vs NHW tumor	2.04	**2.2E-02**	**3.9E-02**	9.29	**3.5E-10**	**1.1E-09**	2.73	1.3E-01	5.8E-01	12.70	**1.0E-15**	**1.0E-15**
18:1	NHW unin. vs NHW tumor	0.03	9.8E-01	6.9E-01	0.22	8.7E-01	5.5E-01	-0.26	8.8E-01	9.4E-01	0.30	8.1E-01	1.0E+00
18:0	NHW unin. vs NHW tumor	0.38	6.7E-01	5.9E-01	0.22	8.8E-01	5.5E-01	-1.72	3.3E-01	8.3E-01	0.00	1.0E+00	1.0E+00
20:0	NHW unin. vs NHW tumor	0.75	4.0E-01	5.5E-01	0.26	8.5E-01	5.5E-01	-0.54	7.6E-01	9.4E-01	0.09	9.4E-01	1.0E+00
22:0	NHW unin. vs NHW tumor	3.81	**2.4E-05**	**5.6E-05**	3.69	**9.5E-03**	**1.5E-02**	3.82	**3.2E-02**	2.3E-01	0.72	5.5E-01	1.0E+00
24:1	NHW unin. vs NHW tumor	9.80	**1.0E-15**	**1.0E-15**	4.51	**1.6E-03**	**3.4E-03**	8.56	**2.6E-06**	**2.3E-05**	2.75	**2.3E-02**	1.7E-01
24:0	NHW unin. vs NHW tumor	10.57	**1.0E-15**	**1.0E-15**	12.91	**1.0E-15**	**1.0E-15**	10.77	**5.5E-09**	**5.5E-08**	2.92	**1.6E-02**	1.4E-01
26:1	NHW unin. vs NHW tumor	0.54	5.4E-01	5.5E-01	0.34	8.1E-01	5.5E-01	1.29	4.7E-01	8.5E-01	0.07	9.5E-01	1.0E+00
26:0	NHW unin. vs NHW tumor	0.56	5.2E-01	5.5E-01	0.78	5.8E-01	5.5E-01	1.84	3.0E-01	8.3E-01	0.03	9.8E-01	1.0E+00

	EEC NHW	sphingoid bases		
species	***comparison***	***means diff.***	***p***	***p-adj. (q)***
d18:1 So	NHW unin. vs NHW tumor	104.62	**6.1E-09**	**2.5E-08**
d18:0 Sa	NHW unin. vs NHW tumor	15.13	**1.4E-04**	**4.1E-04**
d18:1 So1P	NHW unin. vs NHW tumor	1.80	6.4E-01	8.7E-01
d18:0 Sa1P	NHW unin. vs NHW tumor	1.46	6.8E-01	8.7E-01

Supplemental Table S12. Uncorrected (p) and FDR/BKY-adjusted (Q=0.05) p-values (q) for comparisons of unin. vs tumor tissues from NHW females with EEC. Means diff. values that are positive indicate that the corresponding lipid species is higher in the tumor than in the unin. tissue. The lower limit of p- and q-value calculations were set to 1.0E-15 (Prism), so no values are reported lower than this limit. Alterations considered significant (α ≤ 0.05, q ≤ 0.05) are in bold text. Means differences (means diff.) were calculated following ANOVA analysis (Prism).

	COAD NHW	ceramide			monohexosylceramide			sphingomyelin			lactosylceramide		
acyl chain-length	***comparison***	***means diff.***	***p***	***p-adj. (q)***	***means diff.***	***p***	***p-adj. (q)***	***means diff.***	***p***	***p-adj. (q)***	***means diff.***	***p***	***p-adj. (q)***
14:0	NHW unin. vs NHW tumor	0.17	8.1E-01	7.3E-01	0.11	8.9E-01	7.3E-01	2.12	1.4E-01	4.8E-01	0.12	9.3E-01	9.4E-01
16:0	NHW unin. vs NHW tumor	2.07	**4.1E-03**	**1.0E-02**	4.47	**2.3E-08**	**8.5E-08**	1.25	3.8E-01	7.4E-01	13.15	**1.0E-15**	**1.0E-15**
18:1	NHW unin. vs NHW tumor	0.00	1.0E+00	7.3E-01	0.05	9.5E-01	7.3E-01	-0.45	7.5E-01	9.7E-01	-0.05	9.7E-01	9.4E-01
18:0	NHW unin. vs NHW tumor	-0.06	9.4E-01	7.3E-01	-0.02	9.8E-01	7.3E-01	-3.86	**6.9E-03**	7.2E-02	0.04	9.8E-01	9.4E-01
20:0	NHW unin. vs NHW tumor	-0.02	9.8E-01	7.3E-01	0.01	9.9E-01	7.3E-01	-3.38	**1.8E-02**	9.3E-02	0.01	1.0E+00	9.4E-01
22:0	NHW unin. vs NHW tumor	1.13	1.2E-01	2.2E-01	1.52	5.4E-02	1.0E-01	-1.22	3.9E-01	7.4E-01	0.74	5.6E-01	9.4E-01
24:1	NHW unin. vs NHW tumor	4.90	**3.1E-11**	**2.2E-10**	2.33	**3.2E-03**	**7.8E-03**	1.15	4.2E-01	7.4E-01	2.72	**3.2E-02**	1.5E-01
24:0	NHW unin. vs NHW tumor	3.61	**7.4E-07**	**2.7E-06**	6.52	**1.0E-15**	**9.0E-15**	0.60	6.7E-01	9.7E-01	2.13	9.2E-02	2.9E-01
26:1	NHW unin. vs NHW tumor	0.11	8.7E-01	7.3E-01	0.13	8.7E-01	7.3E-01	-0.15	9.2E-01	9.7E-01	0.08	9.5E-01	9.4E-01
26:0	NHW unin. vs NHW tumor	0.20	7.9E-01	7.3E-01	0.31	6.9E-01	7.3E-01	0.13	9.2E-01	9.7E-01	0.00	1.0E+00	9.4E-01

	COAD NHW	sphingoid bases		
species	***comparison***	***means diff.***	***p***	***p-adj. (q)***
d18:1 So	NHW unin. vs NHW tumor	5.34	**1.1E-02**	**1.2E-02**
d18:0 Sa	NHW unin. vs NHW tumor	7.40	**1.6E-03**	**3.3E-03**
d18:1 So1P	NHW unin. vs NHW tumor	0.02	9.9E-01	7.0E-01
d18:0 Sa1P	NHW unin. vs NHW tumor	-0.01	1.0E+00	7.0E-01

Supplemental Table S13. Uncorrected (p) and FDR/BKY-adjusted (Q=5%) p-values (q) for comparisons of unin. vs tumor tissues from NHW males with COAD. Means diff. values that are positive indicate that the corresponding lipid species is higher in the tumor than in the unin. tissue. The lower limit of p- and q-value calculations were set to 1.0E-15 (Prism), so no values are reported lower than this limit. Alterations considered significant ($\alpha \leq 0.05$, $q \leq 0.05$) are in bold text. Means differences (means diff.) were calculated following ANOVA analysis (Prism).

acyl chain-length	HCC NHW comparison	ceramide means diff.	ceramide p	ceramide p-adj. (q)	monohexosylceramide means diff.	monohexosylceramide p	monohexosylceramide p-adj. (q)	sphingomyelin means diff.	sphingomyelin p	sphingomyelin p-adj. (q)	lactosylceramide means diff.	lactosylceramide p	lactosylceramide p-adj. (q)
14:0	NHW unin. vs NHW tumor	0.14	9.5E-01	1.0E+00	0.01	9.9E-01	8.4E-01	1.83	2.6E-01	6.8E-01	0.00	1.0E+00	1.0E+00
16:0	NHW unin. vs NHW tumor	1.84	4.3E-01	1.0E+00	0.44	7.3E-01	8.4E-01	0.53	7.4E-01	1.0E+00	0.09	9.5E-01	1.0E+00
18:1	NHW unin. vs NHW tumor	0.87	7.1E-01	1.0E+00	0.02	9.9E-01	8.4E-01	0.18	9.1E-01	1.0E+00	0.38	7.9E-01	1.0E+00
18:0	NHW unin. vs NHW tumor	0.12	9.6E-01	1.0E+00	-0.01	9.9E-01	8.4E-01	0.06	9.7E-01	1.0E+00	0.04	9.7E-01	1.0E+00
20:0	NHW unin. vs NHW tumor	-0.44	8.5E-01	1.0E+00	-0.06	9.6E-01	8.4E-01	-2.49	1.2E-01	6.8E-01	-0.06	9.6E-01	1.0E+00
22:0	NHW unin. vs NHW tumor	-2.05	3.8E-01	1.0E+00	-3.87	**3.1E-03**	**1.3E-02**	-1.09	5.0E-01	1.0E+00	-2.58	6.6E-02	7.0E-01
24:1	NHW unin. vs NHW tumor	6.57	**5.6E-03**	5.9E-02	0.08	9.5E-01	8.4E-01	2.16	1.8E-01	6.8E-01	-0.88	5.3E-01	1.0E+00
24:0	NHW unin. vs NHW tumor	-5.72	**1.6E-02**	**8.3E-02**	-4.57	**5.0E-04**	**4.2E-03**	1.84	2.5E-01	6.8E-01	-1.13	4.2E-01	1.0E+00
26:1	NHW unin. vs NHW tumor	0.28	9.1E-01	1.0E+00	0.07	9.6E-01	8.4E-01	0.34	8.3E-01	1.0E+00	0.00	1.0E+00	1.0E+00
26:0	NHW unin. vs NHW tumor	0.16	9.5E-01	1.0E+00	0.10	9.4E-01	8.4E-01	-0.37	8.2E-01	1.0E+00	0.00	1.0E+00	1.0E+00

species	HCC NHW comparison	sphingoid bases means diff.	p	p-adj. (q)
d18:1 So	NHW unin. vs NHW tumor	-7.29	**4.5E-02**	1.9E-01
d18:0 Sa	NHW unin. vs NHW tumor	-3.09	7.5E-02	2.4E-01
d18:1 So1P	NHW unin. vs NHW tumor	-0.14	9.3E-02	1.9E-01
d18:0 Sa1P	NHW unin. vs NHW tumor	0.00	1.0E+00	1.0E+00

Supplemental Table S14. Uncorrected (p) and FDR/BKY-adjusted (Q=0.05) p-values (q) for comparisons of unin. vs tumor tissues from NHW males with HCC. Means difference (means diff.) values that are positive indicate that the corresponding lipid species is higher in the tumor than in the unin. tissue. The lower limit of p- and q-value calculations were set to 1.0E-15 (Prism), so no values are reported lower than this limit. Alterations considered significant (α ≤ 0.05, q ≤ 0.05) are in bold text. Means differences were calculated following ANOVA analysis (Prism).

	HNSCC NHW	ceramide			monohexosylceramide			sphingomyelin			lactosylceramide		
acyl chain-length	***comparison***	***means diff.***	***p***	***p-adj. (q)***	***means diff.***	***p***	***p-adj. (q)***	***means diff.***	***p***	***p-adj. (q)***	***means diff.***	***p***	***p-adj. (q)***
14:0	NHW unin. vs NHW tumor	0.41	7.5E-01	6.1E-01	0.01	1.0E+00	1.0E+00	2.79	5.1E-01	8.2E-01	0.32	9.4E-01	7.3E-01
16:0	NHW unin. vs NHW tumor	6.01	**4.7E-06**	**1.6E-05**	0.15	9.4E-01	1.0E+00	1.57	7.1E-01	8.2E-01	29.17	**1.8E-09**	**1.3E-08**
18:1	NHW unin. vs NHW tumor	0.12	9.2E-01	6.8E-01	0.00	1.0E+00	1.0E+00	-2.45	5.6E-01	8.2E-01	0.12	9.8E-01	7.3E-01
18:0	NHW unin. vs NHW tumor	0.50	7.0E-01	6.1E-01	0.15	9.3E-01	1.0E+00	-4.86	2.5E-01	8.2E-01	0.25	9.6E-01	7.3E-01
20:0	NHW unin. vs NHW tumor	1.05	4.1E-01	5.1E-01	0.11	9.5E-01	1.0E+00	-3.15	4.5E-01	8.2E-01	0.04	9.9E-01	7.3E-01
22:0	NHW unin. vs NHW tumor	2.43	5.9E-02	8.6E-02	-3.50	6.0E-02	6.3E-01	-1.18	7.8E-01	8.2E-01	3.95	4.0E-01	7.3E-01
24:1	NHW unin. vs NHW tumor	2.92	**2.3E-02**	**4.3E-02**	0.68	7.1E-01	1.0E+00	1.36	7.5E-01	8.2E-01	22.64	**2.1E-06**	**7.8E-06**
24:0	NHW unin. vs NHW tumor	8.27	**6.8E-10**	**5.0E-09**	0.36	8.5E-01	1.0E+00	2.53	5.5E-01	8.2E-01	12.42	**8.1E-03**	**2.0E-02**
26:1	NHW unin. vs NHW tumor	0.65	6.1E-01	6.1E-01	0.94	6.1E-01	1.0E+00	6.36	1.3E-01	8.2E-01	0.50	9.1E-01	7.3E-01
26:0	NHW unin. vs NHW tumor	5.92	**6.5E-06**	**1.6E-05**	0.84	6.5E-01	1.0E+00	1.23	7.7E-01	8.2E-01	-0.64	8.9E-01	7.3E-01

	HNSCC NHW	sphingoid bases		
species	***comparison***	***means diff.***	***p***	***p-adj. (q)***
d18:1 So	NHW unin. vs NHW tumor	-15.74	1.2E-01	5.1E-01
d18:0 Sa	NHW unin. vs NHW tumor	0.90	8.2E-01	1.0E+00
d18:1 So1P	NHW unin. vs NHW tumor	-0.85	2.0E-01	4.1E-01
d18:0 Sa1P	NHW unin. vs NHW tumor	0.03	9.7E-01	1.0E+00

Supplemental Table S15. Uncorrected (p) and FDR/BKY-adjusted (Q=0.05) p-values (q) for comparisons of unin. vs tumor tissues from NHW males with HNSCC. Means differences (means diff.) values that are positive indicate that the corresponding lipid species is higher in the tumor than in the unin. tissue. The lower limit of p- and q-value calculations were set to 1.0E-15 (Prism), so no values are reported lower than this limit. Alterations considered significant ($\alpha \leq 0.05$, $q \leq 0.05$) are in bold text. Means differences were calculated following ANOVA analysis (Prism).

LUAD		ceramide			monohexosylceramide			sphingomyelin			lactosylceramide		
acyl chain-length	***comparison***	***means diff.***	***p***	***p-adj. (q)***	***means diff.***	***p***	***p-adj. (q)***	***means diff.***	***p***	***p-adj. (q)***	***means diff.***	***p***	***p-adj. (q)***
14:0	AA unin. vs. NHW unin.	-0.02	9.9E-01	1.0E+00	0.00	1.0E+00	1.0E+00	1.74	7.2E-01	1.0E+00	-0.02	9.8E-01	1.0E+00
	AA tumor vs. NHW tumor	-0.09	9.4E-01	1.0E+00	0.01	9.9E-01	1.0E+00	1.35	7.7E-01	1.0E+00	-0.11	9.2E-01	1.0E+00
16:0	AA unin. vs. NHW unin.	0.05	9.7E-01	8.4E-01	-0.63	5.0E-01	3.5E-01	7.12	1.5E-01	4.8E-01	-3.17	**9.7E-03**	**1.0E-02**
	AA tumor vs. NHW tumor	-1.68	1.5E-01	2.6E-01	-1.16	1.8E-01	1.5E-01	0.32	9.4E-01	9.9E-01	-2.65	**2.0E-02**	**1.4E-02**
18:1	AA unin. vs. NHW unin.	0.07	9.5E-01	1.0E+00	-0.03	9.8E-01	1.0E+00	4.17	3.9E-01	6.3E-01	0.00	1.0E+00	1.0E+00
	AA tumor vs. NHW tumor	-0.02	9.9E-01	1.0E+00	-0.08	9.2E-01	1.0E+00	4.20	3.6E-01	6.3E-01	-0.04	9.7E-01	1.0E+00
18:0	AA unin. vs. NHW unin.	-0.34	7.8E-01	1.0E+00	-0.01	9.9E-01	1.0E+00	10.79	**2.8E-02**	1.1E-01	-0.01	9.9E-01	1.0E+00
	AA tumor vs. NHW tumor	-0.55	6.3E-01	1.0E+00	-0.10	9.1E-01	1.0E+00	8.10	7.6E-02	1.2E-01	-0.12	9.2E-01	1.0E+00
20:0	AA unin. vs. NHW unin.	0.03	9.8E-01	1.0E+00	-0.09	9.3E-01	1.0E+00	4.98	3.1E-01	6.2E-01	-0.02	9.8E-01	1.0E+00
	AA tumor vs. NHW tumor	-0.22	8.5E-01	1.0E+00	-0.07	9.4E-01	1.0E+00	7.61	9.6E-02	3.0E-01	-0.08	9.4E-01	1.0E+00
22:0	AA unin. vs. NHW unin.	-0.41	7.4E-01	7.8E-01	-0.55	5.6E-01	7.4E-01	11.18	**2.3E-02**	1.4E-01	-0.40	7.4E-01	9.3E-01
	AA tumor vs. NHW tumor	-0.84	4.7E-01	6.9E-01	-0.17	8.5E-01	8.9E-01	3.93	3.9E-01	4.8E-01	-0.71	5.3E-01	9.3E-01
24:1	AA unin. vs. NHW unin.	-0.02	9.9E-01	1.7E-01	-0.17	8.5E-01	3.0E-01	6.72	1.7E-01	5.4E-01	-1.50	2.2E-01	3.5E-01
	AA tumor vs. NHW tumor	-3.07	**8.2E-03**	**1.7E-03**	0.21	8.1E-01	3.0E-01	4.25	3.5E-01	6.1E-01	-2.25	**4.8E-02**	1.5E-01
24:0	AA unin. vs. NHW unin.	0.66	5.9E-01	3.1E-01	-0.60	5.2E-01	2.2E-01	4.52	3.6E-01	6.7E-01	-0.92	4.5E-01	7.1E-01
	AA tumor vs. NHW tumor	-1.67	1.5E-01	9.5E-02	-0.05	9.6E-01	3.4E-01	4.10	3.7E-01	6.7E-01	-1.91	9.3E-02	2.4E-01
26:1	AA unin. vs. NHW unin.	0.15	9.0E-01	9.8E-01	-0.01	9.9E-01	1.0E+00	1.86	7.0E-01	9.9E-01	-0.01	9.9E-01	1.0E+00
	AA tumor vs. NHW tumor	-0.11	9.2E-01	9.8E-01	0.06	9.5E-01	1.0E+00	1.32	7.7E-01	9.9E-01	-0.06	9.6E-01	1.0E+00
26:0	AA unin. vs. NHW unin.	0.02	9.9E-01	1.0E+00	0.00	1.0E+00	1.0E+00	0.96	8.4E-01	1.0E+00	0.00	1.0E+00	1.0E+00
	AA tumor vs. NHW tumor	-0.10	9.3E-01	1.0E+00	-0.01	9.9E-01	1.0E+00	1.42	7.6E-01	1.0E+00	-0.02	9.9E-01	1.0E+00

	LUAD		LCBs	
species	***comparison***	***means diff.***	***p***	***p-adj. (q)***
d18:1 So	AA unin. vs. NHW unin.	-1.00	3.8E-01	3.3E-01
	AA tumor vs. NHW tumor	-3.41	**1.3E-03**	**6.7E-03**
d18:0 Sa	AA unin. vs. NHW unin.	-0.66	7.5E-02	9.8E-02
	AA tumor vs. NHW tumor	-0.97	**5.1E-03**	**2.7E-02**
d18:1 So1P	AA unin. vs. NHW unin.	0.00	9.6E-01	1.0E+00
	AA tumor vs. NHW tumor	0.01	7.0E-01	8.8E-01
d18:0 Sa1P	AA unin. vs. NHW unin.	0.00	9.5E-01	9.9E-01
	AA tumor vs. NHW tumor	-0.01	6.8E-01	9.9E-01

Supplemental Table S16. Uncorrected (p) and FDR/BKY-adjusted (Q=0.05) p-values (q) for comparisons between AA unin. vs NHW unin. and AA tumor vs NHW tumor tissues of males with LUAD. Means difference (means diff.) positive values indicate levels of the corresponding lipid are higher in the tissues of AA, whereas means diff. negative values indicate levels of the corresponding lipid species are lower in the tissues of AA. The lower limit of p- and q-value calculations were set to 1.0E-15 (Prism), so no values are reported lower than this limit. Alterations considered significant (α ≤ 0.05, q ≤ 0.05) are in bold text. Means differences were calculated following ANOVA analysis (Prism).

	EEC	ceramide			monohexosylceramide			sphingomyelin			lactosylceramide		
acyl chain-length	***comparison***	***means diff.***	***p***	***p-adj. (q)***	***means diff.***	***p***	***p-adj. (q)***	***means diff.***	***p***	***p-adj. (q)***	***means diff.***	***p***	***p-adj. (q)***
14:0	AA unin. vs. NHW unin.	-0.01	9.9E-01	1.0E+00	-0.05	9.7E-01	9.7E-01	-1.51	3.5E-01	2.2E-01	-0.03	9.8E-01	1.0E+00
	AA tumor vs. NHW tumor	-0.06	9.5E-01	1.0E+00	-0.10	9.4E-01	9.7E-01	1.19	4.7E-01	2.5E-01	0.12	9.2E-01	1.0E+00
16:0	AA unin. vs. NHW unin.	0.02	9.8E-01	1.0E+00	-0.87	4.7E-01	4.7E-01	-0.54	7.4E-01	7.7E-01	-0.81	5.2E-01	9.0E-02
	AA tumor vs. NHW tumor	-0.78	4.0E-01	5.0E-01	-4.80	**1.1E-04**	**1.6E-04**	1.02	5.5E-01	6.9E-01	-2.77	**2.9E-02**	**6.0E-03**
18:1	AA unin. vs. NHW unin.	0.00	1.0E+00	1.0E+00	-0.08	9.4E-01	9.5E-01	-0.09	9.6E-01	1.0E+00	-0.06	9.6E-01	1.0E+00
	AA tumor vs. NHW tumor	-0.02	9.8E-01	1.0E+00	-0.15	9.1E-01	9.5E-01	0.06	9.7E-01	1.0E+00	-0.02	9.9E-01	1.0E+00
18:0	AA unin. vs. NHW unin.	-0.01	9.9E-01	1.0E+00	-0.11	9.3E-01	9.3E-01	-1.07	5.1E-01	7.3E-01	-0.10	9.3E-01	1.0E+00
	AA tumor vs. NHW tumor	-0.13	8.9E-01	1.0E+00	-0.11	9.3E-01	9.3E-01	-0.87	6.0E-01	7.3E-01	0.09	9.4E-01	1.0E+00
20:0	AA unin. vs. NHW unin.	-0.06	9.5E-01	1.0E+00	-0.18	8.8E-01	9.9E-01	-1.27	4.3E-01	9.8E-01	-0.02	9.9E-01	1.0E+00
	AA tumor vs. NHW tumor	-0.12	8.9E-01	1.0E+00	0.02	9.9E-01	9.9E-01	0.07	9.6E-01	1.0E+00	0.06	9.6E-01	1.0E+00
22:0	AA unin. vs. NHW unin.	-0.42	6.4E-01	2.2E-01	-0.88	4.6E-01	4.6E-01	-1.25	4.4E-01	2.8E-01	-0.14	9.1E-01	9.5E-01
	AA tumor vs. NHW tumor	-0.67	4.6E-01	1.9E-01	-1.18	3.4E-01	4.0E-01	-1.04	5.3E-01	2.8E-01	0.18	8.8E-01	9.5E-01
24:1	AA unin. vs. NHW unin.	-1.18	1.9E-01	**3.3E-02**	-0.97	4.2E-01	5.1E-01	-1.29	4.3E-01	7.4E-02	-0.43	7.3E-01	7.6E-01
	AA tumor vs. NHW tumor	-4.03	**1.5E-05**	**3.2E-06**	-0.79	5.2E-01	5.2E-01	-3.68	**2.6E-02**	**5.5E-03**	-0.86	5.0E-01	6.2E-01
24:0	AA unin. vs. NHW unin.	0.19	8.3E-01	2.9E-01	-1.30	2.8E-01	2.8E-01	-0.60	7.1E-01	2.5E-01	-0.15	9.0E-01	3.2E-01
	AA tumor vs. NHW tumor	1.03	2.7E-01	1.1E-01	-1.67	1.7E-01	2.1E-01	-3.11	6.0E-02	**2.5E-02**	1.38	2.7E-01	1.1E-01
26:1	AA unin. vs. NHW unin.	-0.01	9.9E-01	1.0E+00	-0.04	9.7E-01	9.7E-01	-0.07	9.7E-01	1.0E+00	-0.01	9.9E-01	1.0E+00
	AA tumor vs. NHW tumor	-0.25	7.8E-01	9.9E-01	-0.16	9.0E-01	9.7E-01	-0.47	7.8E-01	9.8E-01	-0.03	9.8E-01	1.0E+00
26:0	AA unin. vs. NHW unin.	0.00	1.0E+00	1.0E+00	-0.02	9.9E-01	9.9E-01	0.05	9.8E-01	1.0E+00	0.00	1.0E+00	1.0E+00
	AA tumor vs. NHW tumor	-0.40	6.7E-01	1.0E+00	-0.50	6.8E-01	9.8E-01	-0.87	6.0E-01	7.5E-01	-0.01	9.9E-01	1.0E+00

	EEC	LCBs		
species	***comparison***	***means diff.***	***p***	***p-adj. (q)***
d18:1 So	AA unin. vs. NHW unin.	-5.83	9.0E-01	1.6E-01
	AA tumor vs. NHW tumor	134.84	**4.4E-03**	**1.5E-03**
d18:0 Sa	AA unin. vs. NHW unin.	0.65	8.5E-01	3.0E-01
	AA tumor vs. NHW tumor	-1.05	7.5E-01	3.0E-01
d18:1 So1P	AA unin. vs. NHW unin.	-0.22	8.4E-01	8.8E-01
	AA tumor vs. NHW tumor	-1.19	2.9E-01	6.1E-01
d18:0 Sa1P	AA unin. vs. NHW unin.	-0.27	7.9E-01	9.8E-01
	AA tumor vs. NHW tumor	-1.55	1.4E-01	3.0E-01

Supplemental Table S17. Uncorrected (p) and FDR/BKY-adjusted (Q=0.05) p-values (q) for comparisons between AA unin. vs NHW unin. and AA tumor vs NHW tumor tissues of females with EEC. Means difference (means diff.) positive values indicate levels of the corresponding lipid are higher in the tissues of AA, whereas means diff. negative values indicate levels of the corresponding lipid species are lower in the tissues of AA. The lower limit of p- and q-value calculations were set to 1.0E-15 (Prism), so no values are reported lower than this limit. Alterations considered significant (α ≤ 0.05, q ≤ 0.05) are in bold text. Means differences were calculated following ANOVA analysis (Prism).

COAD		ceramide			monohexosylceramide			sphingomyelin			lactosylceramide		
acyl chain-length	***comparison***	***means diff.***	***p***	***p-adj. (q)***	***means diff.***	***p***	***p-adj. (q)***	***means diff.***	***p***	***p-adj. (q)***	***means diff.***	***p***	***p-adj. (q)***
14:0	AA unin. vs. NHW unin.	0.02	9.9E-01	1.0E+00	0.03	9.7E-01	1.0E+00	-0.80	6.0E-01	7.6E-01	-0.04	9.8E-01	1.0E+00
	AA tumor vs. NHW tumor	0.07	9.4E-01	1.0E+00	0.19	8.1E-01	1.0E+00	0.40	8.0E-01	8.4E-01	-0.08	9.6E-01	1.0E+00
16:0	AA unin. vs. NHW unin.	0.61	5.4E-01	3.8E-01	0.73	3.6E-01	1.3E-01	-1.29	4.0E-01	3.1E-01	1.23	4.2E-01	7.4E-02
	AA tumor vs. NHW tumor	0.92	3.5E-01	3.0E-01	1.42	7.4E-02	**3.1E-02**	3.02	**4.9E-02**	6.8E-02	6.16	**5.7E-05**	**1.2E-05**
18:1	AA unin. vs. NHW unin.	0.03	9.8E-01	1.0E+00	0.11	8.9E-01	1.0E+00	-0.10	9.5E-01	1.0E+00	-0.08	9.6E-01	1.0E+00
	AA tumor vs. NHW tumor	0.05	9.6E-01	1.0E+00	0.19	8.1E-01	1.0E+00	0.05	9.8E-01	1.0E+00	0.03	9.8E-01	1.0E+00
18:0	AA unin. vs. NHW unin.	0.02	9.9E-01	1.0E+00	0.07	9.3E-01	1.0E+00	-0.15	9.2E-01	3.2E-01	0.04	9.8E-01	1.0E+00
	AA tumor vs. NHW tumor	0.11	9.1E-01	1.0E+00	0.10	9.0E-01	1.0E+00	-0.22	8.9E-01	3.2E-01	0.07	9.6E-01	1.0E+00
20:0	AA unin. vs. NHW unin.	0.02	9.9E-01	1.0E+00	0.01	9.9E-01	1.0E+00	-0.43	7.8E-01	8.2E-01	-0.01	1.0E+00	1.0E+00
	AA tumor vs. NHW tumor	0.04	9.7E-01	1.0E+00	-0.01	9.9E-01	1.0E+00	-0.44	7.8E-01	8.2E-01	0.00	1.0E+00	1.0E+00
22:0	AA unin. vs. NHW unin.	0.18	8.6E-01	9.0E-01	0.49	5.4E-01	6.8E-01	0.68	6.6E-01	7.1E-01	0.01	9.9E-01	1.0E+00
	AA tumor vs. NHW tumor	0.25	8.0E-01	9.0E-01	-0.13	8.7E-01	9.1E-01	-0.64	6.8E-01	7.1E-01	0.11	9.4E-01	1.0E+00
24:1	AA unin. vs. NHW unin.	4.97	**6.8E-07**	**1.8E-07**	0.71	3.7E-01	2.3E-01	0.61	6.9E-01	9.1E-01	-0.06	9.7E-01	1.0E+00
	AA tumor vs. NHW tumor	9.14	**1.0E-15**	**1.0E-15**	0.59	4.6E-01	2.4E-01	0.04	9.8E-01	1.0E+00	0.91	5.5E-01	6.9E-01
24:0	AA unin. vs. NHW unin.	0.61	5.4E-01	9.5E-02	1.98	**1.3E-02**	**2.8E-03**	0.58	7.0E-01	8.9E-01	-0.22	8.9E-01	9.6E-01
	AA tumor vs. NHW tumor	2.25	**2.4E-02**	**5.1E-03**	1.29	1.1E-01	**1.9E-02**	1.06	4.9E-01	8.9E-01	0.16	9.1E-01	9.6E-01
26:1	AA unin. vs. NHW unin.	0.17	8.7E-01	1.0E+00	0.03	9.7E-01	1.0E+00	-0.01	1.0E+00	1.0E+00	-0.01	1.0E+00	1.0E+00
	AA tumor vs. NHW tumor	0.40	6.9E-01	1.0E+00	0.04	9.6E-01	1.0E+00	0.05	9.7E-01	1.0E+00	-0.01	1.0E+00	1.0E+00
26:0	AA unin. vs. NHW unin.	0.03	9.8E-01	1.0E+00	0.08	9.2E-01	9.7E-01	0.06	9.7E-01	1.0E+00	0.00	1.0E+00	1.0E+00
	AA tumor vs. NHW tumor	0.01	9.9E-01	1.0E+00	-0.07	9.3E-01	9.7E-01	-0.12	9.4E-01	1.0E+00	0.02	9.9E-01	1.0E+00

COAD		LCBs		
species	***comparison***	***means diff.***	***p***	***p-adj. (q)***
d18:1 So	AA unin. vs. NHW unin.	-0.55	7.7E-01	4.1E-01
	AA tumor vs. NHW tumor	-1.62	4.0E-01	2.5E-01
d18:0 Sa	AA unin. vs. NHW unin.	-6.03	**4.6E-03**	**9.8E-04**
	AA tumor vs. NHW tumor	-6.84	**1.4E-03**	**3.6E-04**
d18:1 So1P	AA unin. vs. NHW unin.	0.00	9.8E-01	1.0E+00
	AA tumor vs. NHW tumor	0.02	7.9E-01	1.0E+00
d18:0 Sa1P	AA unin. vs. NHW unin.	-0.08	3.9E-01	6.0E-01
	AA tumor vs. NHW tumor	0.10	2.9E-01	6.0E-01

Supplemental Table S18. Uncorrected (p) and FDR/BKY-adjusted (Q=0.05) p-values (q) for comparisons between AA unin. vs NHW unin. and AA tumor vs NHW tumor tissues of males with COAD. Means difference (means diff.) positive values indicate levels of the corresponding lipid are higher in the tissues of AA, whereas means diff. negative values indicate levels of the corresponding lipid species are lower in the tissues of AA. The lower limit of p- and q-value calculations were set to 1.0E-15 (Prism), so no values are reported lower than this limit. Alterations considered significant (α ≤ 0.05, q ≤ 0.05) are in bold text. Means differences were calculated following ANOVA analysis (Prism).

	HCC	ceramide			monohexosylceramide			sphingomyelin			lactosylceramide		
acyl chain-length	***comparison***	***means diff.***	***p***	***p-adj. (q)***	***means diff.***	***p***	***p-adj. (q)***	***means diff.***	***p***	***p-adj. (q)***	***means diff.***	***p***	***p-adj. (q)***
14:0	AA unin. vs. NHW unin.	0.06	9.9E-01	1.0E+00	-0.02	9.9E-01	1.0E+00	2.80	4.3E-01	8.2E-01	0.08	9.6E-01	1.0E+00
	AA tumor vs. NHW tumor	0.05	9.9E-01	1.0E+00	-0.02	9.9E-01	1.0E+00	-3.25	3.6E-01	8.2E-01	-0.07	9.6E-01	1.0E+00
16:0	AA unin. vs. NHW unin.	0.54	8.8E-01	9.2E-01	0.17	8.9E-01	9.5E-01	4.67	1.8E-01	7.1E-01	4.78	**2.2E-03**	**3.5E-03**
	AA tumor vs. NHW tumor	-0.57	8.7E-01	9.2E-01	-0.58	6.2E-01	9.5E-01	2.65	4.5E-01	7.1E-01	-0.83	6.0E-01	4.0E-01
18:1	AA unin. vs. NHW unin.	0.03	9.9E-01	1.0E+00	0.22	8.5E-01	1.0E+00	-0.09	9.8E-01	1.0E+00	0.41	7.9E-01	1.0E+00
	AA tumor vs. NHW tumor	-0.76	8.3E-01	1.0E+00	0.18	8.8E-01	1.0E+00	-0.22	9.5E-01	1.0E+00	-0.24	8.8E-01	1.0E+00
18:0	AA unin. vs. NHW unin.	0.10	9.8E-01	1.0E+00	0.01	1.0E+00	1.0E+00	0.40	9.1E-01	1.0E+00	0.04	9.8E-01	1.0E+00
	AA tumor vs. NHW tumor	-0.25	9.5E-01	1.0E+00	0.00	1.0E+00	1.0E+00	-0.78	8.2E-01	1.0E+00	0.07	9.7E-01	1.0E+00
20:0	AA unin. vs. NHW unin.	-0.10	9.8E-01	1.0E+00	-0.01	9.9E-01	1.0E+00	-0.07	9.8E-01	1.0E+00	0.08	9.6E-01	1.0E+00
	AA tumor vs. NHW tumor	-0.19	9.6E-01	1.0E+00	0.03	9.8E-01	1.0E+00	-1.09	7.6E-01	9.5E-01	-0.01	1.0E+00	1.0E+00
22:0	AA unin. vs. NHW unin.	1.72	6.3E-01	6.9E-01	-2.81	**1.7E-02**	**1.8E-02**	3.79	2.8E-01	4.1E-01	-0.28	8.6E-01	9.0E-01
	AA tumor vs. NHW tumor	5.43	1.3E-01	6.9E-01	0.29	8.0E-01	4.2E-01	-3.47	3.2E-01	4.1E-01	-0.38	8.1E-01	9.0E-01
24:1	AA unin. vs. NHW unin.	11.77	**1.2E-03**	**2.5E-03**	0.10	9.4E-01	1.0E+00	5.56	1.1E-01	3.6E-01	0.81	6.0E-01	8.5E-01
	AA tumor vs. NHW tumor	7.21	**4.6E-02**	6.4E-02	-0.05	9.7E-01	1.0E+00	-4.75	1.8E-01	3.7E-01	0.51	7.5E-01	8.5E-01
24:0	AA unin. vs. NHW unin.	-2.16	5.5E-01	4.8E-01	-1.53	1.9E-01	8.1E-02	7.38	**3.7E-02**	1.2E-01	0.19	9.0E-01	9.5E-01
	AA tumor vs. NHW tumor	11.14	**2.1E-03**	**1.1E-02**	0.04	9.7E-01	3.4E-01	-2.80	4.3E-01	6.7E-01	-1.58	3.2E-01	5.9E-01
	AA unin. vs. NHW unin.	0.04	9.9E-01	1.0E+00	0.00	1.0E+00	1.0E+00	0.11	9.8E-01	1.0E+00	0.01	9.9E-01	1.0E+00
26:1	AA tumor vs. NHW tumor	0.24	9.5E-01	1.0E+00	-0.01	9.9E-01	1.0E+00	-0.38	9.1E-01	1.0E+00	0.00	1.0E+00	1.0E+00
26:0	AA unin. vs. NHW unin.	-0.03	9.9E-01	1.0E+00	-0.03	9.8E-01	1.0E+00	-0.12	9.7E-01	1.0E+00	0.01	9.9E-01	1.0E+00
	AA tumor vs. NHW tumor	-0.15	9.7E-01	1.0E+00	-0.15	9.0E-01	1.0E+00	-0.29	9.4E-01	1.0E+00	-0.01	1.0E+00	1.0E+00

	HCC	LCBs		
species	***comparison***	***means diff.***	***p***	***p-adj. (q)***
d18:1 So	AA unin. vs. NHW unin.	9.11	**4.3E-02**	7.6E-02
	AA tumor vs. NHW tumor	6.15	1.7E-01	1.8E-01
d18:0 Sa	AA unin. vs. NHW unin.	8.08	**7.7E-04**	**9.9E-04**
	AA tumor vs. NHW tumor	3.05	1.9E-01	1.2E-01
d18:1 So1P	AA unin. vs. NHW unin.	-0.16	9.7E-01	9.7E-01
	AA tumor vs. NHW tumor	-0.07	9.9E-01	9.9E-01
d18:0 Sa1P	AA unin. vs. NHW unin.	0.00	1.0E+00	1.0E+00
	AA tumor vs. NHW tumor	0.00	1.0E+00	1.0E+00

Supplemental Table S19. Uncorrected (p) and FDR/BKY-adjusted (Q=0.05) p-values (q) for comparisons between AA unin. vs NHW unin. and AA tumor vs NHW tumor tissues of males with HCC. Means difference (means diff.) positive values indicate levels of the corresponding lipid are higher in the tissues of AA, whereas means diff. negative values indicate levels of the corresponding lipid species are lower in the tissues of AA. The lower limit of p- and q-value calculations were set to 1.0E-15 (Prism), so no values are reported lower than this limit. Alterations considered significant (α ≤ 0.05, q ≤ 0.05) are in bold text. Means differences were calculated following ANOVA analysis (Prism).

	HNSCC	ceramide			monohexosylceramide			sphingomyelin			lactosylceramide		
acyl chain-length	***comparison***	***means diff.***	***p***	***p-adj. (q)***	***means diff.***	***p***	***p-adj. (q)***	***means diff.***	***p***	***p-adj. (q)***	***means diff.***	***p***	***p-adj. (q)***
14:0	AA unin. vs. NHW unin.	-0.09	9.5E-01	1.0E+00	-0.08	9.7E-01	1.0E+00	-6.38	1.6E-01	3.3E-01	-0.10	9.8E-01	1.0E+00
	AA tumor vs. NHW tumor	0.62	7.0E-01	1.0E+00	0.20	9.3E-01	1.0E+00	-1.32	7.8E-01	8.1E-01	-0.10	9.8E-01	1.0E+00
16:0	AA unin. vs. NHW unin.	0.17	9.1E-01	1.6E-01	-2.53	2.8E-01	1.8E-01	8.58	5.7E-02	2.1E-01	-9.56	**2.9E-02**	**6.1E-03**
	AA tumor vs. NHW tumor	3.47	**3.2E-02**	**6.8E-03**	8.70	**3.4E-04**	**3.6E-04**	-1.47	7.5E-01	9.5E-01	8.73	5.1E-02	**9.0E-03**
18:1	AA unin. vs. NHW unin.	0.00	1.0E+00	1.0E+00	0.00	1.0E+00	1.0E+00	-1.34	7.7E-01	1.0E+00	-0.04	9.9E-01	1.0E+00
	AA tumor vs. NHW tumor	-0.10	9.5E-01	1.0E+00	0.03	9.9E-01	1.0E+00	0.04	9.9E-01	1.0E+00	0.11	9.8E-01	1.0E+00
18:0	AA unin. vs. NHW unin.	-0.01	1.0E+00	1.0E+00	0.05	9.8E-01	1.0E+00	2.55	5.7E-01	7.2E-01	-0.35	9.4E-01	1.0E+00
	AA tumor vs. NHW tumor	0.78	6.3E-01	9.5E-01	0.77	7.5E-01	1.0E+00	-0.02	1.0E+00	1.0E+00	0.14	9.7E-01	1.0E+00
20:0	AA unin. vs. NHW unin.	-0.05	9.7E-01	1.0E+00	0.00	1.0E+00	1.0E+00	-3.00	5.1E-01	1.0E+00	-0.10	9.8E-01	1.0E+00
	AA tumor vs. NHW tumor	1.92	2.4E-01	5.0E-01	1.43	5.5E-01	1.0E+00	0.61	8.9E-01	1.0E+00	0.23	9.6E-01	1.0E+00
22:0	AA unin. vs. NHW unin.	-0.54	7.3E-01	5.1E-01	-0.13	9.6E-01	1.0E+00	-3.44	4.5E-01	1.0E+00	-1.33	7.6E-01	9.6E-01
	AA tumor vs. NHW tumor	1.60	3.2E-01	2.7E-01	5.27	**2.9E-02**	1.8E-01	-0.02	1.0E+00	1.0E+00	0.07	9.9E-01	1.0E+00
24:1	AA unin. vs. NHW unin.	-2.07	1.9E-01	1.0E-01	0.84	7.2E-01	4.9E-01	0.85	8.5E-01	1.0E+00	-5.73	1.9E-01	8.0E-02
	AA tumor vs. NHW tumor	2.17	1.8E-01	1.0E-01	6.14	**1.1E-02**	**1.4E-02**	-0.17	9.7E-01	1.0E+00	-5.34	2.3E-01	8.1E-02
24:0	AA unin. vs. NHW unin.	-0.78	6.2E-01	1.1E-01	-1.59	5.0E-01	3.2E-01	-2.21	6.2E-01	6.6E-01	-4.64	2.9E-01	1.2E-01
	AA tumor vs. NHW tumor	5.39	**9.5E-04**	**2.0E-04**	10.40	**2.0E-05**	**2.1E-05**	2.71	5.6E-01	6.6E-01	-2.60	5.6E-01	2.0E-01
26:1	AA unin. vs. NHW unin.	-0.11	9.4E-01	9.9E-01	0.18	9.4E-01	9.9E-01	0.20	9.6E-01	1.0E+00	-0.90	8.4E-01	1.0E+00
	AA tumor vs. NHW tumor	0.60	7.1E-01	8.9E-01	4.44	6.6E-02	1.4E-01	0.04	9.9E-01	1.0E+00	-0.30	9.5E-01	1.0E+00
26:0	AA unin. vs. NHW unin.	-0.19	9.0E-01	3.2E-01	1.93	4.1E-01	8.3E-01	-0.10	9.8E-01	1.0E+00	-1.26	7.7E-01	1.0E+00
	AA tumor vs. NHW tumor	1.43	3.8E-01	1.6E-01	1.72	4.8E-01	8.3E-01	0.20	9.7E-01	1.0E+00	0.41	9.3E-01	1.0E+00

	HNSCC		LCBs	
species	**comparison**	***means diff.***	***p***	***p-adj. (q)***
d18:1 So	AA unin. vs. NHW unin.	-13.99	7.5E-02	1.6E-01
	AA tumor vs. NHW tumor	-4.00	6.2E-01	7.8E-01
d18:0 Sa	AA unin. vs. NHW unin.	-0.27	9.4E-01	9.9E-01
	AA tumor vs. NHW tumor	-4.61	2.1E-01	7.3E-01
d18:1 So1P	AA unin. vs. NHW unin.	-0.84	7.5E-02	1.6E-01
	AA tumor vs. NHW tumor	-0.12	8.0E-01	1.0E+00
d18:0 Sa1P	AA unin. vs. NHW unin.	0.02	9.7E-01	1.0E+00
	AA tumor vs. NHW tumor	-0.03	9.5E-01	1.0E+00

Supplemental Table S20. Uncorrected (p) and FDR/BKY-adjusted (Q=0.05) p-values (q) for comparisons between AA unin. vs NHW unin. and AA tumor vs NHW tumor tissues of males with HNSCC. Means difference (means diff.) positive values indicate levels of the corresponding lipid are higher in the tissues of AA, whereas means diff. negative values indicate levels of the corresponding lipid species are lower in the tissues of AA. The lower limit of p- and q-value calculations were set to 1.0E-15 (Prism), so no values are reported lower than this limit. Alterations considered significant (α ≤ 0.05, q ≤ 0.05) are in bold text. Means differences were calculated following ANOVA analysis (Prism).

	Pan-cancer	ceramide			monohexosylceramide			sphingomyelin			lactosylceramide		
acyl chain-length	***comparison***	***means diff.***	***p***	***p-adj. (q)***	***means diff.***	***p***	***p-adj. (q)***	***means diff.***	***p***	***p-adj. (q)***	***means diff.***	***p***	***p-adj. (q)***
14:0	unin. vs tumor	0.3	6.6E-01	5.1E-01	0.2	7.1E-01	6.3E-01	1.9	1.2E-01	2.5E-01	0.1	8.9E-01	7.2E-01
16:0	unin. vs tumor	2.8	**1.3E-06**	**2.6E-06**	4.1	**1.0E-15**	**1.0E-15**	2.1	8.4E-02	2.5E-01	12.9	**1.0E-15**	**1.0E-15**
18:1	unin. vs tumor	0.1	8.8E-01	5.5E-01	0.1	8.6E-01	6.3E-01	-0.4	7.6E-01	8.8E-01	0.1	9.1E-01	7.2E-01
18:0	unin. vs tumor	0.2	7.3E-01	5.1E-01	0.1	8.1E-01	6.3E-01	-2.1	8.8E-02	2.5E-01	0.1	8.6E-01	7.2E-01
20:0	unin. vs tumor	0.4	5.0E-01	4.5E-01	0.2	7.2E-01	6.3E-01	-0.9	4.4E-01	6.6E-01	0.0	9.5E-01	7.2E-01
22:0	unin. vs tumor	1.7	**2.6E-03**	**4.1E-03**	0.6	1.6E-01	2.7E-01	-0.2	8.4E-01	8.8E-01	0.8	3.1E-01	5.6E-01
24:1	unin. vs tumor	6.6	**1.0E-15**	**1.0E-15**	3.4	**2.0E-15**	**4.0E-15**	1.9	1.2E-01	2.5E-01	5.0	**4.2E-10**	**1.5E-09**
24:0	unin. vs tumor	5.7	**1.0E-15**	**1.0E-15**	4.9	**1.0E-15**	**1.0E-15**	2.3	6.0E-02	2.5E-01	3.3	**3.9E-05**	**9.6E-05**
26:1	unin. vs tumor	0.4	5.0E-01	4.5E-01	0.6	1.8E-01	2.7E-01	0.9	4.3E-01	6.6E-01	0.2	8.4E-01	7.2E-01
26:0	unin. vs tumor	1.1	**4.9E-02**	6.2E-02	0.2	5.6E-01	6.3E-01	0.4	7.6E-01	8.8E-01	0.0	9.7E-01	7.2E-01

	Pan-cancer	sphingoid bases		
species	***comparison***	***means diff.***	***p***	***p-adj. (q)***
d18:1 So	unin. vs tumor	17.54	**6.2E-05**	**2.0E-04**
d18:0 Sa	unin. vs tumor	2.14	**4.6E-03**	**9.7E-03**
d18:1 So1P	unin. vs tumor	0.07	5.0E-01	5.3E-01
d18:0 Sa1P	unin. vs tumor	0.12	2.2E-01	4.7E-01

Supplemental Table S21. Uncorrected (p) and FDR/BKY-adjusted (Q=0.05) p-values (q) for comparisons using data pooled from AA and NHW subjects with LUAD, EEC, COAD, and HNSCC. Comparisons were made between unin. vs tumor tissues. Means diff. values that are positive indicate that the corresponding lipid species is higher in the tumor than in the unin. tissue. The lower limit of p- and q-value calculations were set to 1.0E-15 (Prism), so no values are reported lower than this limit. Alterations considered significant (α ≤ 0.05, q ≤ 0.05) are in bold text. Means differences were calculated following ANOVA analysis (Prism).

acyl chain-length	Pan-cancer comparison	ceramide means diff.	ceramide p	ceramide p-adj. (q)	monohexosylceramide means diff.	monohexosylceramide p	monohexosylceramide p-adj. (q)	sphingomyelin means diff.	sphingomyelin p	sphingomyelin p-adj. (q)	lactosylceramide means diff.	lactosylceramide p	lactosylceramide p-adj. (q)
14:0	AA unin. vs. NHW unin.	0.00	1.0E+00	1.0E+00	0.00	1.0E+00	1.0E+00	0.31	8.6E-01	9.0E-01	-0.03	9.8E-01	1.0E+00
	AA tumor vs. NHW tumor	0.10	9.0E-01	1.0E+00	0.11	8.6E-01	1.0E+00	1.37	4.2E-01	6.6E-01	-0.06	9.6E-01	1.0E+00
16:0	AA unin. vs. NHW unin.	0.31	7.1E-01	2.7E-01	-0.07	9.1E-01	1.6E-01	1.90	2.7E-01	5.6E-01	-1.16	3.1E-01	5.3E-02
	AA tumor vs. NHW tumor	0.23	7.7E-01	2.7E-01	1.26	**3.5E-02**	**7.3E-03**	0.20	9.1E-01	9.5E-01	3.55	**1.6E-03**	**3.4E-04**
18:1	AA unin. vs. NHW unin.	0.03	9.7E-01	1.0E+00	0.06	9.2E-01	1.0E+00	0.52	7.6E-01	9.3E-01	0.03	9.8E-01	1.0E+00
	AA tumor vs. NHW tumor	-0.11	9.0E-01	1.0E+00	0.07	9.1E-01	1.0E+00	0.75	6.6E-01	9.3E-01	0.00	1.0E+00	1.0E+00
18:0	AA unin. vs. NHW unin.	-0.10	9.0E-01	9.5E-01	0.01	9.8E-01	1.0E+00	1.12	5.2E-01	6.5E-01	-0.05	9.7E-01	1.0E+00
	AA tumor vs. NHW tumor	-0.11	9.0E-01	9.5E-01	0.09	8.8E-01	1.0E+00	-0.58	7.3E-01	7.7E-01	0.02	9.8E-01	1.0E+00
20:0	AA unin. vs. NHW unin.	-0.06	9.4E-01	9.9E-01	-0.05	9.3E-01	9.9E-01	-1.19	4.9E-01	8.9E-01	-0.02	9.9E-01	1.0E+00
	AA tumor vs. NHW tumor	0.12	8.8E-01	9.9E-01	0.18	7.6E-01	9.9E-01	-0.68	6.9E-01	8.9E-01	0.01	9.9E-01	1.0E+00
22:0	AA unin. vs. NHW unin.	-0.15	8.6E-01	9.0E-01	-0.51	4.0E-01	5.1E-01	0.87	6.1E-01	8.2E-01	-0.35	7.5E-01	9.5E-01
	AA tumor vs. NHW tumor	0.51	5.3E-01	6.6E-01	0.49	4.1E-01	5.1E-01	-1.81	2.9E-01	8.2E-01	-0.13	9.1E-01	9.5E-01
24:1	AA unin. vs. NHW unin.	3.25	**7.5E-05**	**8.8E-05**	0.10	8.7E-01	3.1E-01	1.14	5.1E-01	6.4E-01	-1.17	3.0E-01	1.3E-01
	AA tumor vs. NHW tumor	3.54	**1.3E-05**	**2.1E-05**	0.41	4.9E-01	2.1E-01	-1.80	2.9E-01	6.0E-01	-0.93	4.1E-01	1.4E-01
24:0	AA unin. vs. NHW unin.	-0.61	4.6E-01	8.0E-02	0.22	7.2E-01	1.3E-01	0.57	7.4E-01	8.5E-01	-1.02	3.7E-01	2.3E-01
	AA tumor vs. NHW tumor	2.33	**4.0E-03**	**8.5E-04**	2.26	**1.5E-04**	**3.2E-05**	-0.42	8.1E-01	8.5E-01	-0.76	5.0E-01	2.6E-01
26:1	AA unin. vs. NHW unin.	0.09	9.1E-01	9.5E-01	0.06	9.2E-01	9.7E-01	0.21	9.0E-01	1.0E+00	-0.13	9.1E-01	1.0E+00
	AA tumor vs. NHW tumor	0.18	8.2E-01	9.5E-01	0.65	2.8E-01	5.8E-01	0.00	1.0E+00	1.0E+00	-0.06	9.6E-01	1.0E+00
26:0	AA unin. vs. NHW unin.	-0.02	9.8E-01	1.0E+00	0.37	5.4E-01	1.0E+00	-0.01	9.9E-01	1.0E+00	-0.17	8.8E-01	1.0E+00
	AA tumor vs. NHW tumor	0.12	8.8E-01	1.0E+00	0.18	7.7E-01	1.0E+00	-0.05	9.8E-01	1.0E+00	0.06	9.6E-01	1.0E+00

species	Pan-cancer comparison	LCBs means diff.	LCBs p	LCBs p-adj. (q)
d18:1 So	AA unin. vs. NHW unin.	-1.20	8.5E-01	4.4E-01
	AA tumor vs. NHW tumor	16.64	**7.0E-03**	**7.4E-03**
d18:0 Sa	AA unin. vs. NHW unin.	0.72	5.0E-01	4.4E-01
	AA tumor vs. NHW tumor	-0.79	4.6E-01	4.4E-01
d18:1 So1P	AA unin. vs. NHW unin.	-0.18	2.1E-01	5.0E-01
	AA tumor vs. NHW tumor	-0.17	2.4E-01	5.0E-01
d18:0 Sa1P	AA unin. vs. NHW unin.	-0.05	7.3E-01	9.1E-01
	AA tumor vs. NHW tumor	-0.14	3.2E-01	6.7E-01

Supplemental Table S22. Uncorrected (p) and FDR/BKY-adjusted (Q=0.05) p-values (q) for comparisons using data pooled from subjects with LUAD, EEC, COAD, and HNSCC. Comparisons were made between AA unin. vs NHW unin. and AA tumor vs NHW tumor tissues. Means difference (means diff.) positive values indicate levels of the corresponding lipid are higher in the tissues of AA, whereas means diff. negative values indicate levels of the corresponding lipid species are lower in the tissues of AA. The lower limit of p- and q-value calculations were set to 1.0E-15 (Prism), so no values are reported lower than this limit. Alterations considered significant (α ≤ 0.05, q ≤ 0.05) are in bold text. Means differences were calculated following ANOVA analysis (Prism).
